# Patterns of Sympathetic Responses Induced by Different Stress Tasks

**DOI:** 10.2174/1874205X00802010025

**Published:** 2008-05-19

**Authors:** M Fechir, T Schlereth, T Purat, S Kritzmann, C Geber, T Eberle, M Gamer, F Birklein

**Affiliations:** 1 Department of Neurology, University of Mainz, Langenbeckstrasse 1, 55101 Mainz, Germany; 2 Department of Systems Neuroscience, University Medical Center Hamburg-Eppendorf, Martinistr. 52, 20248 Hamburg, Germany; 3 Department of Physiology, University of Mainz, Duesbergweg 6, 55128 Mainz, Germany

## Abstract

Stress tasks are used to induce sympathetic nervous system (SNS) arousal. However, the efficacy and the patterns of SNS activation have not been systematically compared between different tasks.

Therefore, we analyzed SNS activation during the following stress tasks: Presentation of negative, positive, and – as a control – neutral affective pictures, Color-Word interference test (CWT), mental arithmetic under time limit, singing a song aloud, and giving a spontaneous talk. We examined 11 healthy subjects and recorded the following SNS parameters: Activation of emotional sweating by quantitative sudometry, skin vasoconstriction by laser-Doppler flowmetry, heart rate by ECG, blood pressure by determination of pulse wave transit time (PWTT), and electromyographic (EMG) activity of the trapezius muscle. Moreover, subjective stress ratings were acquired for each task using a visual analog scale.

All tasks were felt significantly stressful when compared to viewing neutral pictures. However, SNS activation was not reliable: Affective pictures did not induce a significant SNS response; singing, giving a talk and mental arithmetic selectively increased heart rate and emotional sweating. Only the CWT globally activated the SNS. Regarding all tasks, induction of emotional sweating, increase of heart rate and blood pressure significantly correlated with subjective stress ratings, in contrast to EMG and skin vasoconstriction.

Our results show that the activation of the SNS widely varies depending on the stress task. Different stress tasks differently activate the SNS, which is an important finding when considering sympathetic reactions - in clinical situations and in research.

## INTRODUCTION

Sympathetic activation (e.g. by stress) is part of our daily life. An excessive activity of the sympathetic nervous system (SNS) might underlie different stress-related disorders. It is important to notice that these stress-related disorders are diverse; they might be serious like hypertension [1], annoying like stress-related myalgic pain [2, 3], or harmless like hyperhidrosis or flushing [4]. In contrast differential activation of the SNS in the lab is usually neglected. Only the difference between thermoregulatory and emotional SNS activation is commonly accepted. Thermoregulatory activation (e.g. by cold environment) activates vasoconstriction but inhibits sweating while emotional stimuli activate both. Only few studies have addressed the emotional activation of the SNS in detail.

Standard emotional stimuli which have been used in experimental studies to induce SNS activation comprise the international affective picture system (IAPS [5]) assembly [6, 7]. It has been shown that IAPS-related changes of SNS activity depend on valence of the pictures [8] and on general levels of individual anxiety [9]. Therefore, gender-specific versions of the picture system have been generated [10]. The Color-Word interference test (CWT) [11] is another task which has been repeatedly used in stress research [12-15]. In previous studies it has been shown that task pacing (self-paced vs. automatically paced), task speed [16], and response mode affected SNS activation [17]. Less common stress tasks are forcing participants to accomplish mental arithmetic under time limit [18, 19], singing a song aloud or giving a spontaneous talk to an audience [20, 21].

Undoubtedly, all these tasks induce SNS activation in general. However, it is less clear whether they differentially activate specific subcomponents of the SNS (e.g. blood pressure, heart rate, sweating). In the present study, we therefore analysed SNS activation in detail during different tasks. A finding of task-dependent SNS activation patterns would demonstrate that stress responses might be more specific than commonly assumed. This study was designed not only to improve our understanding of SNS function but also to facilitate comprehension and comparability of different studies focussing on SNS activation.

## SUBJECTS AND METHODS

### Subjects

We examined 11 young healthy volunteers (median age 24 years, range 22-29 years; 5 men, 6 women). None of them had a history of previous autonomic disease (e.g. diabetes mellitus) or took any medication which might influence the autonomic nervous system. Moreover, there was no evidence of hypertension in any subject. All subjects abstained from smoking and drinking caffeine on the day of investigation. Informed consent was obtained from all subjects and the study adhered to the tenets of the Declaration of Helsinki. The study was approved by the local ethics committee.

Volunteers rested in a supine position and were asked to perform the different stress tasks as described below. Room temperature (24°C) and humidity (50% relative humidity) were controlled, all subjects acclimatised for one hour. At the end of acclimatisation, skin temperature at the finger tips was measured using an infrared thermometer and was found to be above 30°C in any subject.

### Sympathetic Arousals (Stress Tasks)

24 affective pictures with negative valence and 24 pictures with positive valence were selected from the international affective picture system (IAPS) [5] (see appendix for numbers of IAPS). The picture assemblies were different for men and women. 24 neutral pictures served as control. The pictures were presented with a duration of five seconds for each picture using a Powerpoint® slide show. Since spontaneous arousal while viewing neutral pictures might be better controlled than during baseline condition, SNS parameters of all stress tasks were compared to viewing neutral pictures in a separate analysis (see below).

The Color-Word interference test (CWT) used in our study was automatically paced. Color words written in a different color were presented on a PC screen every 2 seconds and the subjects had to indicate the color of the presented word (not the meaning of the word) verbally. Responses were automatically recorded.

The mental arithmetic task consisted of a visually presented subtraction calculation exercise with a multiple choice solution. Response was given verbally.

As a fourth task, subjects had to give a spontaneous talk to the lab personnel for two minutes. The topic of the talk (i.e. death penalty) was previously unknown.

Finally, all subjects had to sing a German children’s song aloud to the examiners. The text was presented in words, singing was also performed for two minutes.

### Sympathetic Activation Parameters

Cardio–vascular parameters were registered with a FAN-device (Schwarzer, Germany). We extracted heart rate and pulse wave transit time (PWTT) from the FAN data. PWTT is a surrogate for arterial blood pressure [22]. The analysis of parameters indicating heart rate variability was inappropriate since 1) recording time was too short and 2) these parameters are often affected by breathing. Intentional breathing changes occurred during talking or singing, but this does not necessarily reflect autonomic activation.

Emotional sweating was measured using quantitative hygrometry [23]. In brief, sweat capsules were affixed to palms and constantly streamed by dry nitrogen. Humidity from sweat glands was measured downstream by capacitance hygrometry. For quantification, curves were intregrated, the area under the curve (AUC) for the respective period was computed and compared to the AUC of a baseline [23].

Surface electromyography (EMG) of the trapezius muscle was recorded by a Pathway MR-20-EMG (Prometheus Group, Dover, New Hampshire, USA). Electrodes were placed at 2/3 of the distance between vertebra prominens and acromion. Employing Synergy 3-D software (The Prometheus Group, Dover, New Hampshire, USA) EMG signals were rectified and then integrated to yield EMG sum activity.

Peripheral vasoconstriction was determined by measuring skin blood flow at the fingertips of the second finger employing the single point mode of a laser Doppler imager (LDI, Moor Instruments Limited, London, UK) as previously described [24]. In order to avoid movement artefacts, the forearm was fixed in a splint. Sampling frequency of the LDI was 20 Hz, time constant was set to 0.1 s, and distance to skin was 50 cm. Laser signals were further processed using dedicated software (moorLDI SPM 3.01; Moor Instruments, London, UK) and expressed as flux values (FV).

As all of these parameters regularly show fluctuations depending on the actual state of arousal, we computed difference values between the stress tasks, which lasted 120 seconds, and the mean values during a baseline period of 30 seconds before each single task.

Special care was taken that all sympathetic parameters returned to baseline between subsequent tasks.

In order to exclude differences of fitness, personality, and hormonal status, all known to affect SNS activation, we focussed on within-subject variability of SNS response. Thereby, influence of individual variability was minimized.

### Study Design

The stress tasks were presented to the subjects in a randomised order; each task was performed only once in order to avoid adaptation. Choosing a randomised order we avoided the possibility of a higher state of arousal at the beginning of the experiment. The duration of each task was exactly two minutes. Between the tasks, participants were allowed to rest for at least five minutes. Directly after each stress task subjects rated subjective stressfulness of the preceding task on a 10 cm visual analog scale ranging from no (0) to maximal imaginable stress (10).

Examination was performed by MF and TP. TP recruited the subjects and knew them, which reduced unspecific arousal.

### Statistical Analysis

Statistical analyses were performed using the software package “SPSS 12.0 for windows” (SPSS Inc., Chicago, Illinois, USA). In the first analyses, we compared differences between baseline and SNS responses of the different stress tasks. For this purpose we used a within-subject ANOVA and post-hoc *t*-tests. The same type of ANOVA was employed to compare the different tasks to viewing “neutral” pictures, which was defined as the control condition in our study. Correlation between mean variables was assessed by computing Spearman rank order correlations. Data are presented as mean +/- standard error of the mean (SEM). Statistical significance was assumed for p < 0.05.

## RESULTS

### 1 Stress Ratings

Presentation of negative affective pictures led to significantly increased stress awareness as compared to neutral pictures (4.5 +/- 0.7 vs 2.0 +/- 0.4 cm VAS; p < 0.01). Subjects even judged the positive pictures to be more “stressful” than the neutral ones (3.6 +/- 0.5 vs 2.0 +/- 0.4 cm VAS; p < 0.05).

All other tasks induced significantly higher stress than viewing neutral affective pictures, too. Singing was rated 4.3 +/- 0.7 cm VAS (p < 0.05), CWT 5.4 +/- 0.5 cm VAS (p < 0.01), talking 6.5 cm +/- 0.6 cm VAS (p < 0.01) and mental arithmetic 7.3 +/- 0.6 cm VAS (p < 0.01). For detail and statistics see Fig. (**1**).

### 2 Emotional Sweating

Presentation of neutral, negative and positive affective pictures did not significantly activate emotional sweating at palms when compared to baseline (neutral pictures 127 AUC +/- 17 vs baseline 126 AUC +/- 17, n.s.; negative pictures 125 AUC +/- 13 vs baseline 122 AUC +/- 13, ns; positive pictures 124 AUC +/- 14 vs baseline 121 AUC +/- 13, ns).

In contrast to that, CWT (299 AUC +/- 36 vs baseline 268 AUC +/- 37, p < 0.01), mental arithmetic (312 AUC +/- 31 vs baseline 284 AUC +/- 32, p < 0.05), singing (144 AUC +/- 20 vs baseline 126 AUC +/- 16, p < 0.05) and giving a talk (147 AUC +/- 19 vs baseline 128 AUC +/- 15, p < 0.05) increased emotional sweating.

Compared to neutral pictures as a reference, sweating did not differ significantly during presentation of negative and positive affective pictures. However, sweat rate was significantly increased during CWT (p < 0.01), mental arithmetic, singing and giving a talk (each p < 0.05). For details see Fig. (**2A**).

### 3 Sympathetic Vasoconstriction

Stress-induced vasoconstriction quickly habituates [4]. Therefore, vasoconstriction was determined as mean flux during the first minute of each task compared to the baseline period (30 sec before). As expected, neutral pictures did not induce vasoconstriction (172 FV +/- 39 vs baseline 201 FV +/- 51, ns). Giving a talk also failed to induce vasoconstriction (91 FV +/- 24 vs baseline 112 FV +/- 31, ns). All other stress tasks, however, evoked significant vasoconstriction when compared to baseline (negative pictures 140 FV +/- 38 vs baseline 187 +/- 56, p < 0.05; positive pictures 180 FV +/- 37 vs baseline 221 FV +/- 48, p <0.05; CWT 124 FV +/- 28 vs baseline 232 FV +/- 59, p < 0.05; mental arithmetic 98 FV +/- 28 vs baseline 149 FV +/- 40, p < 0.05; singing 128 FV +/- 29 vs baseline 187 FV +/- 46, p < 0.05).

Only vasoconstriction during CWT significantly differed from vasoconstriction during neutral pictures viewing (p < 0.05). For details see Fig. (**2B**).

### 4 Cardio-Vascular Parameters

Heart rate did not significantly increase during presentation of neutral (69 bpm +/- 2 vs baseline 69 bpm +/- 2, n.s.), negative (72 bpm +/- 3 vs baseline 70 bpm +/- 3 bpm, n.s.) or positive affective pictures (70 bpm +/- 2 vs baseline 69 bpm +/- 3 bpm, n.s.). All others stress tasks induced heart rate acceleration (CWT 76 bpm +/- 3 vs baseline 70 bpm +/- 4 bmp, p < 0.05; mental arithmetic 75 bpm +/- 2 vs baseline 68 bpm +/- 2 bpm, p < 0.05; singing 74 bpm +/- 2 vs baseline 67 bpm +/-3 bpm, p < 0.01; giving a talk 80 bpm +/- 3 vs baseline 75 bpm +/- 2 bpm, p < 0.05).

Compared to neutral pictures all stress tasks increased heart rate - with the exception of negative and positive pictures (CWT p < 0.05; mental arithmetic p < 0.01; singing p < 0.01, giving a talk p< 0.05). See Fig. (**2C**).

**Fig. (2) All data are presented as mean +/- SEM.**
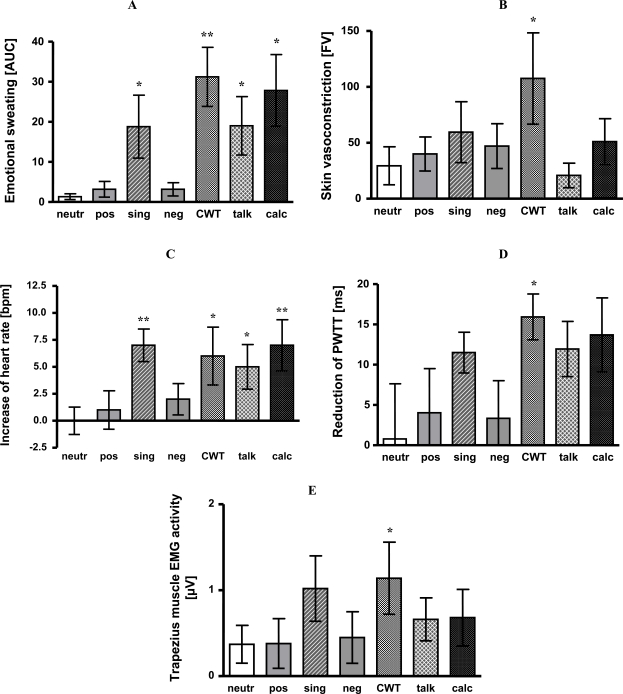
Emotional sweating is expressed as area under the sweating curve (AUC). Singing (p < 0.05), CWT (p <0.01), giving a talk (p <0.05) and mental arithmetic (p < 0.05) induced significant emotional sweating as compared to baseline. Significant differences to neutral affective pictures are indicated by stars. Skin blood flow vasoconstriction (differences of flux values, FV). Compared to baseline, presentation of positive (p < 0.05) and negative pictures (p < 0.05), singing (p < 0.05), CWT (p < 0.05) and mental arithmetic (p < 0.05) led to significant vasoconstriction. Significant differences to neutral affective pictures are indicated by stars. Heart rate increase as compared to baseline (bpm). Singing (p < 0.01), CWT (p < 0.05), giving a talk (p < 0.05) and mental arithmetic (p < 0.01) induced significant increase of heart rate as compared to baseline. Significant differences to neutral affective pictures are indicated by stars. Reduction of PWTT (ms). Singing (p < 0.01), CWT (p < 0.01), giving a talk (p < 0.01) and mental arithmetic (p < 0.05) caused significant reduction of PWTT as compared to baseline. Significant differences to neutral affective pictures are indicated by stars. Activity in trapezius muscle EMG (µV). Singing (p <0.05), CWT (p < 0.05) and giving a talk (p < 0.05) significantly increased trapezius muscle EMG activity as compared to baseline. Significant differences to neutral affective pictures are indicated by stars. neutr: neutral affective pictures; pos: positive affective pictures; sing: singing; neg: negative affective pictures; CWT: Color-Word interference test; talk: giving a talk; calc: mental arithmetic; *: p < 0.05; **: p < 0.01.

Compared to baseline, presentation of affective pictures did not change PWTT. All other tasks shortened PWTT indicating blood pressure increase (CWT 179 ms +/- 5 vs baseline 195 ms +/- 5 ms, p < 0.01; mental arithmetic 180 ms +/- 4 vs baseline 193 ms +/- 4 ms, p < 0.05; singing 179 ms +/- 4 vs baseline 191 ms +/- 4 ms, p < 0.01; giving a talk 180 ms +/- 5 vs baseline 192 ms +/- 4 ms, p < 0.01). Compared to neutral affective pictures only CWT caused a significant additional PWTT shortening (p < 0.05). The other tasks, albeit reducing PWTT, failed to reach significance (Fig. **2D**).

### 5 Trapezius Muscle EMG Activity

Compared to individual baselines, no significant increase of trapezius muscle activity could be observed for presentation of neutral (9.45 µV +/- 0.96 vs baseline 9.08 µV +/- 0.95, ns), negative (10.25 µV +/- 0.86 vs baseline 9.80 µV +/- 0.94, ns) nor positive affective pictures (9.69 µV +/- 0.67 vs baseline 9.31 µV +/- 0.55, ns), nor for mental arithmetic (12.11 µV +/- 0.76 vs baseline 11.43 µV +/- 0.75, ns). In contrast, giving a talk (10.36 µV +/- 0.52 vs baseline 9.70 µV +/- 0.58, p < 0.05), singing (12.37 µV +/- 0.92 vs baseline 11.35 µV +/- 0.5, p < 0.05) and CWT (10.00 µV +/- 0.49 vs baseline 8.86 µV +/- 0.51 µV, p < 0.05) increased trapezius muscular activity.

When compared to neutral pictures, trapezius muscle EMG activity only increased during CWT (p < 0.05) (Fig. (**2E**).

### 6 Correlation Between Subjective Stress Ratings and Sympathetic Activation

In a first step, we correlated the individual stress ratings (n=11 subjects) during all tasks with the different sympathetic parameters. In the whole correlation matrix (7 tasks x 5 parameters) only three correlations were significant: Between stress ratings and PWTT during viewing neutral pictures (r=0.72, p < 0.05), between stress ratings and PWTT during singing (r=0.73, p < 0.05), and between stress ratings and LDI vasoconstriction during giving a talk (r=0.86, p <0.01).

In the second step, we analyzed whether subjective stress affected sympathetic activation on a group level. The mean stress ratings of the different tasks (n=7) were compared to the mean of the sympathetic activation parameters. As shown in Fig. (**3**), activation of emotional sweating (r=0.89, p < 0.01), acceleration of heart rate (r=0.93, p < 0.01) and reduction of PWTT (r=0.79, p < 0.05) significantly correlated with subjective stress ratings. Only trapezius EMG and skin vasoconstriction remained insignificant.

## DISCUSSION

The adaptive stress response counteracts intrinsic or extrinsic threats to homeostasis. Examples are arousal or alertness [25]. One component in the complex concert of human stress response is activation of the sympathetic nervous system (SNS). It is widely believed that SNS activity during stress is changed in a global fashion - by premotor neurons in hypothalamus and brainstem, which induce sympathetic responses in a simultaneous and parallel way [26]. However, if stress becomes chronic, it becomes responsible for a variety of very different diseases and there is no indication for one common “stress disease”. Nevertheless, in the lab, stress tasks are used “uncritically” in order to activate the sympathetic nervous system, for example in imaging studies [27], although there have already been indications that the SNS response might be differentiated [28, 29]. Therefore, the present investigation aimed to assess sympathetic responses in more depth. We found that all common stress tasks under investigation activated the SNS. However, the extent and the pattern of SNS activation varied significantly. Moreover, we also found that only some SNS parameters significantly correlated with stress perception while others did not show a graded response.

The standardization of investigation and within-subject design of our study obviously reduced variability of sympathetic activation. Therefore, we are confident to have identified characteristic intra-individual patterns of SNS activation, even though the number of subjects in our study was small. Nevertheless, this should be taken into account and preclude generalizing our results to different cohorts.

### The Different Components of Sympathetic Activation During Stress Tasks

Our results indicate that the CWT [30], albeit subjectively only moderately stressful, most robustly activates the components of the SNS under investigation. Moreover, the CWT is the only test, which activates the SNS even in addition to viewing neutral IAPS pictures, which serves as “control” condition in our study. This finding is mainly in accordance with previous studies [13, 31], which found the CWT to induce increased heart rate and decreased heart rate variability [17, 32]. Our results indicate that the CWT additionally increases blood pressure, induces emotional sweating and skin vasoconstriction, and activates trapezius muscles. This means, CWT might be the most recommended task when the origin of different SNS components in the brain  e.g. by functional imaging –  are to be investigated in detail. The other tests, even if perceived as more stressful, activated the SNS less reliably. For example, giving a talk did not significantly induce vasoconstriction, mental arithmetic, albeit subjectively most stressful [19], did not increase trapezius muscle activity. When compared to neutral affective pictures, an increase of SNS activity during mental arithmetic was only found for emotional sweating and heart rate.

### IAPS Pictures are Weak SNS Stimuli

Presentation of negative or positive affective pictures is a generally established stress task [33]. However, our results indicate that pictures of both valences induced only minor SNS activation. The only parameter, which was significantly different from baseline was skin vasoconstriction. Moreover, there was no difference of any SNS parameter between neutral and positive or negative affective pictures. This is in contrast to a previous study, which reported changes in skin conductance (a surrogate for emotional sweating) during the presentation of affective pictures [33]. It might be speculated that those pictures evoked higher levels of stress than in our study. Interestingly, affective pictures in the aforementioned study did not induce EMG activity or increase of heart rate, which supports our results.

Likely, the context of presentation affects SNS activation of affective pictures. An increase of heart rate during presentation of affective pictures has been observed - if the subjects focused on the heartbeats [34], or if erotic pictures were shown to men but not to women [35]. The main proportion of the pictures did not induce SNS changes – as in the present study. A more detailed investigation of the SNS response to affective pictures found an initial decrease and a later increase of heart rate during presentation [36]. Since we did not analyse this aspect of SNS changes, we might have missed these minor SNS activations. However, this does not change our assumption that affective pictures only minimally activate the SNS although they are considered as stressful.

### Which SNS Activation Best Reflects Stress?

Perception of stress is complex and varies substantially between individuals [37]. Stress initiates the cognitive evaluation of a situation, it affects behaviour and decision making, induces humoral responses and finally activates the SNS [25]. Perception of SNS activation might subsequently even reinforce stress perception [38]. Given the complexity of the stress response, it is astonishing that we were able to show that subjective perception of stress linearly correlated with heart rate, blood pressure and emotional sweating. This result is in particular important since many functional brain imaging studies investigating the stress response used correlation analysis between stressfulness and brain activation in order to dissect brain areas, for example cognitive evaluation or decision making [39]. Our results now admonish to consider that any brain activation, which is identified by correlation analysis, might be also due to neuronal correlates of SNS activation. Functional imaging studies investigating stress should therefore be carefully controlled – at least with respect to the SNS parameters mentioned above. Muscle and vasoconstrictor activity did not show a graded response as a function of subjective stress perception; they seem to respond in an all-or-nothing fashion. In previous studies, if two categories of subjects or stress tasks have been compared – one category with low and one with high stress ratings – SNS activation was significantly stronger if high stress was perceived [40, 41]. These previous results strongly support our statements.

In conclusion, our study demonstrates that activation of the SNS during stress tasks might vary significantly – depending on the task and the SNS parameter under investigation. Our results favour a more differentiated appreciation of sympathetic reactions – in clinical situations and in research.

## Figures and Tables

**Fig. (1):**
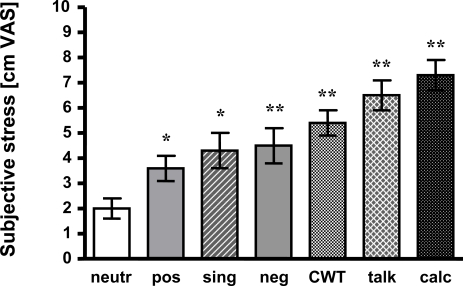
**Mean +/- SEM of subjective stress ratings are presented**. Compared to neutral pictures all tasks induced more stress.neutr: neutral affective pictures; pos: positive affective pictures;sing: singing task; neg: negative affective pictures; CWT: Color-Word interference test; talk: hold a talk; calc: mental arithmetic;*: p < 0.05 vs neutr; **: p < 0.01 vs neutr.

**Fig. (3):**
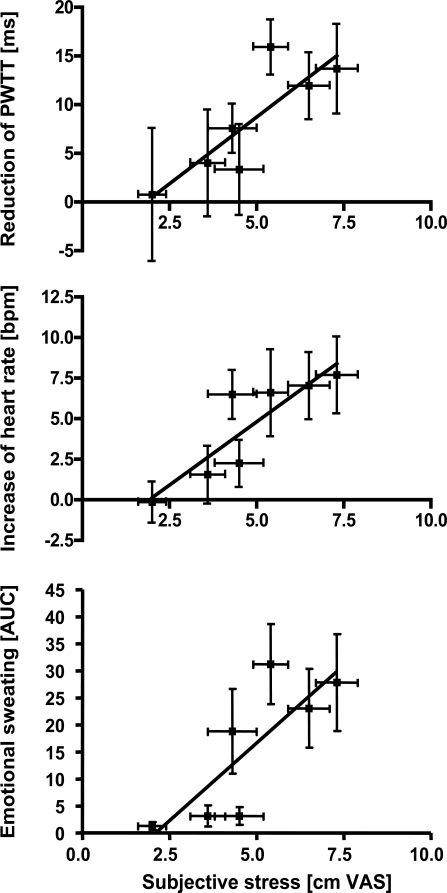
Correlation analyses between mean subjective stress ratings during different tasks and the corresponding mean of sympathetic responses. Stress ratings were significantly correlated to emotional sweating (p < 0.01), increase of heart rate (p < 0.01) and reduction of PWTT (p < 0.05, Spearman rank order correlation).

## Appendix

### The IAPS slide numbers were as follows:

Neutral content, female version: 2200, 2210, 5500, 5510, 7000, 7010, 7020, 7030, 7050, 7060, 2383, 2410, 2520, 5120, 5130, 7009, 7031, 7130, 7150, 2216, 7234, 7700, 7705, 9360.

Neutral content, male version: 2190, 2200, 5500, 7000, 7010, 7020, 7050, 7080, 7090, 7100, 2220, 2230, 5530, 6150, 9070, 7830, 7820, 7620, 7550, 7500, 7190, 7150, 7130, 7110.

Negative content, female version: 1120, 3000, 3053, 3060, 3120, 3170, 3266, 3500, 6300, 6550, 6200, 6210, 6212, 6230, 6313, 6350, 6510, 6540, 9050, 9405, 9570, 9410, 9433, 9400.

Negative content, male version: 3010, 3120, 3530, 9810, 9410, 6350, 3060, 3400, 9921, 9250, 6230, 6250, 6260, 6300, 6313, 6350, 6370, 6510, 6530, 6540, 6550, 6560, 6570, 6821.

Positive content, female version: 4490, 4510, 4660, 4659, 4687, 5621, 7270, 8370, 8490, 2216, 8501, 8496, 8300, 8200, 8190, 8185, 8180, 8161, 8034, 8030, 7502, 4681, 4660, 4658.

Positive content, male version: 4180, 4210, 4290, 4607, 4800, 5621, 5629, 8170, 8200, 8340, 4001, 4002, 4220, 4310, 4608, 4651, 4659, 4664, 8190, 8260, 8370, 8380, 8400, 8420.
